# Management of visual disturbances in albinism: a case report

**DOI:** 10.1186/1752-1947-6-316

**Published:** 2012-09-19

**Authors:** Rokiah Omar, Siti Salwa Idris, Chung Kah Meng, Victor Feizal Knight

**Affiliations:** 1 Optometry and Visual Science Program, School of Healthcare Sciences, Faculty of Health Sciences, Universiti Kebangsaan Malaysia, Jalan Raja Muda Abdul Aziz, 50300, Kuala Lumpur, Malaysia; 2 Chung Optometry Consultant, 2-G-45, Wisma Rampai, Jalan 34/26, Rampai Town Centre, Setapak, 53300, Kuala Lumpur, Malaysia; 3 Faculty of Medicine and Defence Health, National Defence University of Malaysia, Sg Besi Camp, 57000, Kuala Lumpur, Malaysia

**Keywords:** Albino, Low-vision rehabilitation, Special contact lenses, Telemicroscopes

## Abstract

**Introduction:**

A number of vision defects have been reported in association with albinism, such as photophobia, nystagmus and astigmatism. In many cases only prescription sunglasses are prescribed. In this report, the effectiveness of low-vision rehabilitation in albinism, which included prescription of multiple visual aids, is discussed.

**Case presentation:**

We present the case of a 21-year-old Asian woman with albinism and associated vision defects. Her problems were blurring of distant vision, glare and her dissatisfaction with her current auto-focus spectacle-mounted telescope device, which she reported as being heavy as well as cosmetically unacceptable. We describe how low-vision rehabilitation using multiple visual aids, namely spectacles, special iris-tinted contact lenses with clear pupils, and bi-level telemicroscopic apparatus devices improved her quality of life. Subsequent to rehabilitation our patient is happier and continues to use the visual aids.

**Conclusions:**

Contact lenses with a special iris tint and clear pupil area are useful aids to reduce the glare experienced by albinos. Bi-level telemicroscopic apparatus telemicroscopes fitted onto our patient’s prescription spectacles were cosmetically acceptable and able to improve her distance vision. As a result these low-vision rehabilitation approaches improved the quality of life of our albino patient.

## Introduction

Albinism refers to a congenital disorder characterized by a group of conditions that are inherited as a recessive genetic trait [1-3]. Individuals with albinism have either a reduced level of or no pigment in their eyes, skin, or hair. Albinism affects people from all races and it is estimated that the incidence of albinism in the general population is approximately 1:17,000 [2,4]. This incidence appears lowest amongst Asians [4]. Individuals with albinism will always have some form of vision difficulty, which could include refractive error, nystagmus, glare, and tropia. Many have visual dysfunction such that they are classified as being in the category of low vision [5,6]. A survey on the quality of life among albinos was conducted by Omar and Fatin [7] who found that albinos had most difficulties when they were looking at distant objects (100%), with glare (96%), when watching television (60%) and crossing a road alone (50%). It was also found that most albinos were able to read quite well with the aid of magnifiers. The majority of the albinos (76.7%) in the study were able to conduct their daily living activities independently.

In general the quality of life of albinos is lower compared to normal persons mainly due to blurred vision at distance and glare [7]. Many are ‘legally blind’ but a majority are still able to utilize their vision for reading purposes and do not depend on Braille. Individuals with albinism are excellent candidates for low-vision rehabilitation because the visual problems experienced by albinos are usually at the mild to moderate stage and they have stable central vision loss with usually excellent residual side vision [7]. Unfortunately, most albinos only receive limited low-vision aids such as spectacles, sunglasses, monocular telescopes or magnifiers [8] even though there are many types of low-vision aids available in the market. This case report describes the management of a patient with low vision and albinism using a number of low-vision aids such as spectacles, iris- tinted contact lenses with a clear pupil, and the bi-level telemicroscopic apparatus (BITA®) vision enhancer at a Low Vision Clinic in Kuala Lumpur, Malaysia.

## Case presentation

**Figure 1:**
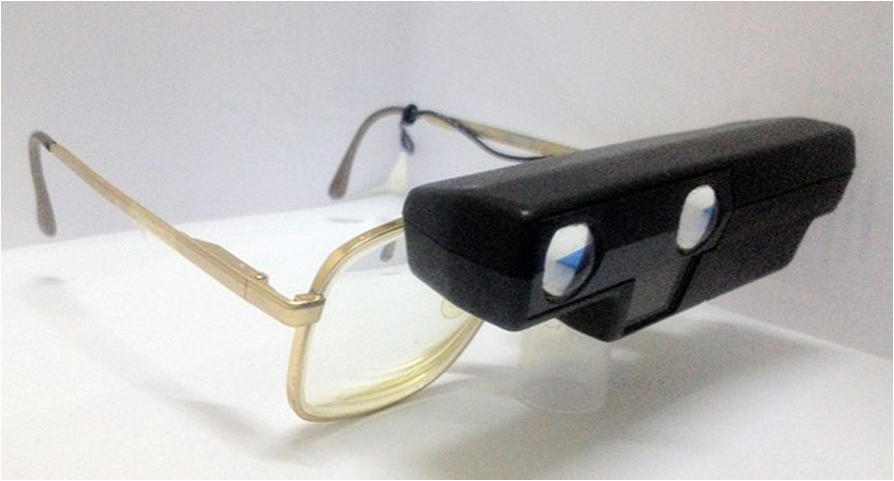
Our patient’s previous autofocus telescope and prescription spectacles.

A 21-year-old Malay woman diagnosed as having albinism presented to a Low Vision Clinic for low-vision assessment. Her problems were blurring of distant vision, glare, and her dissatisfaction with her current auto-focus spectacle-mounted telescope, which she reported as being heavy as well as cosmetically unacceptable (Figure 1). She had poor visual acuity and sensitivity to bright light and glare, and her ocular presentation showed light colored irides, right eye esotropia, pendular nystagmus, and refractive error.

The distance spectacle prescription for her right eye was −7.00/-3.25×170 with visual acuity of 6/48 and for the left eye was −8.00/-2.00×175 with visual acuity of 6/48. Near visual acuity for both right eye and left eye was N10 at 20cm. Subjective refraction was found to be similar to her spectacle prescription on presentation, and after conducting a pinhole test did not show any further improvement.

**Figure 2:**
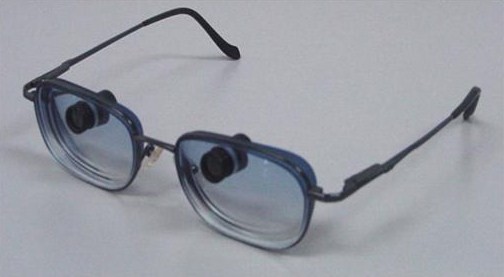
**Bi-level telemicroscopic apparatus (BITA®) telemicroscopes and prescription spectacles, newly prescribed to our patient.**

Low-vision assessment was carried out and the BITA® Vision Enhancer 3/8 4× binocularly was introduced and fitted to her distance spectacles (Figure 2). Our patient found that the BITA® telemicroscope was acceptable and her visual acuity (VA) for both eyes was 6/9 when using the BITA® device. The BITA® telemicroscope was light in weight compared to her auto-focus telescopes. It was also smaller in size and therefore not readily noticeable by others when in use. The BITA® telemicroscope was therefore prescribed to our patient.

**Figure 3:**
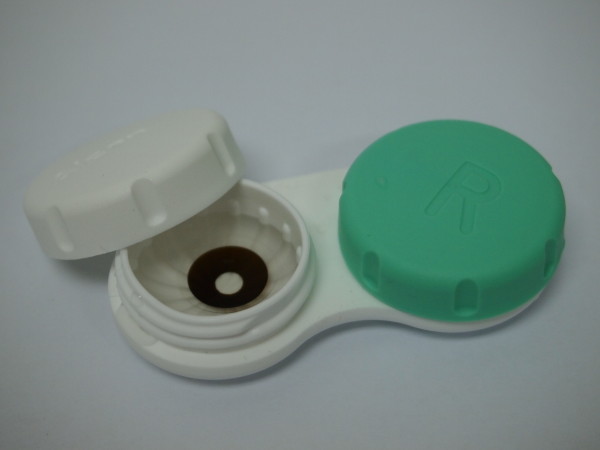
Prosthetic tinted contact lenses with clear pupil area used by our patient.

A slit lamp examination showed that our patient could be fitted with contact lenses (CLs). Her tear prism was sufficient and the tear break-up time (TBUT) was 13 seconds for both eyes. In order to reduce glare, Flexcon prosthetic iris-tinted CM Brown color CLs 38% (12mm iris diameter with 3.5mm clear pupil size) /8.4/14.00/plano/ were prescribed to our patient for both eyes (Figure 3). No prescription was incorporated into the CLs because our patient wanted to use the CLs either with her distance spectacles or with the BITA® telemicroscope. Her vision when using the CLs and her own spectacles improved one line to 6/36 for both eyes. The CLs were prescribed with a multi-purpose solution as the disinfecting system. Our patient returned for aftercare following one week of CL usage. She reported that with the prosthetic CLs, glare was no longer a major issue, her nystagmus was reduced and her vision was clearer with her spectacles. She also reported that her vision was clearer when she used the iris-tinted CLs with the BITA® telemicroscope. Our patient was advised to return for routine CL aftercare every three months, as is advised to other CL wearers.

## Discussion

The issue of glare, sensitivity to light and occurrence of nystagmus in patients with albinism is the result of overstimulation of the hypopigmented retina under normal circumstances. The tinted iris pattern imprinted on the CL reduces the amount of light that enters the eye and thus alleviated our patient’s sensitivity to light and her problem with glare. The reduced light entering her eye, together with the 3.5mm clear pupil on the prescribed CL, forms a smaller blur circle on the fovea hence reducing the scanning that induces our patient’s nystagmus.

Patients with albinism generally have hypopigmentation of the retina, therefore under normal circumstances there is a relative increase in the absorption of light because of the reduced pigment in the retina. As a result, the hypopigmented retina is relatively more stimulated compared to a normally pigmented retina. Hence, a reduction in the light entering the eye and falling onto the hypopigmented retina would cause a subjective improvement in vision, as was described by our patient and demonstrated by her distance vision improving by one Snellen line. The reduced squinting in our patient was observed when she entered a brightly illuminated area while using the CLs. The reduced amount of light entering the eye caused by the use of the CLs delivered a positive benefit to the visual ability of our patient. Similar findings were observed by Philips in a severe albino patients fitted with soft prosthetic contact lens that reduced both the photophobic symptoms and enhanced facial cosmesis [9].

CL aftercare is very important and serves to ensure that the patient’s eyes are closely monitored. Since our patient has nystagmus, complications caused by CL use have to be expected. Frequent CL aftercare appointments are warranted in order to minimize any potential CL complications. Patient education on lens care and maintenance is also needed to ensure that their eyes stay healthy and to avoid any possible eye infection. Consequently, the need for continued CL aftercare was emphasized to our patient so that she could continue using the prosthetic CLs with a minimum of complications.

When the prosthetic CLs were used together with the BITA® telemicroscope, our patient reported that she had clearer vision. The clearer vision could be explained by the improvement in her binocular vision. It is interesting to note that in some cases of nystagmus, blurring of vision in one eye (instead of occluding the eye) will improve the vision of the other eye. The use of the telemicroscope itself does cause some pupillary constriction, thus reducing the light entering the eye and reducing the stimulation of the fovea, hence causing the subjective improvement in vision that our patient reported.

The BITA® telemicroscope is very small (smaller than a paper clip) and much lighter compared to our patient’s old auto-focus telescope. It was therefore not surprising that the BITA® telemicroscope was easily accepted by our patient. This made our patient more confident and more willing to accept using low-vision aids. Ultimately, this combination of management has enabled our patient to cope better with her outdoor activities and to do so in a more productive manner. A previous study has demonstrated that the BITA® telemicroscope is small in size and therefore not generally noticeable to an onlooker. The spectacle-mounted BITA® telemicroscopes allow patients to see a magnified view through the device while simultaneously seeing a normal view through the spectacles [10]. The BITA® telemicroscope is capable of remaining in focus for distances from about three feet to infinity without the need for any adjustment. It can also be adjusted for near work by turning the barrel of each scope to focus. As the telemicroscopes are very small and situated at the upper part of the lenses, they look almost like normal spectacles, as can be seen in Figure 2. A special tint on the spectacle lenses can further camouflage the telescopic device so that it becomes less noticeable to onlookers.

## Conclusions

Contact lenses with special iris tinting and a clear pupil area can be introduced to patients with albinism and can benefit these patients by reducing the amount of light entering the hypopigmented eye, thereby reducing the symptoms of glare and sensitivity to light. However, care and diligence is needed to assess the suitability and usefulness of the tinted contact lenses so that they fulfill a rehabilitative role in patients with albinism. The BITA® telemicroscopes fitted to the prescription spectacles appeared more cosmetically acceptable to our patient, and were able to facilitate our patient in coping with her daily activities thus improving her quality of life through clearer distance vision.

## Consent

Written informed consent was obtained from the patient for publication of this case report and any accompanying images. A copy of the written consent is available for review by the Editor-in-Chief of this journal.

## Competing interests

The authors declare that they have no competing interests.

## Authors’ contributions

RO examined our patient, analyzed and interpreted investigative data and wrote the manuscript. SSI examined and analyzed the investigative data. CKM performed a literature review and contributed to writing the manuscript. VFK made critical revisions and contributed to the manuscript writing. All authors read and approved the final manuscript.
